# Evaluation of Post-Larval Diets for Indoor Weaned Largemouth Bass (*Micropterus salmoides*)

**DOI:** 10.3390/ani13203179

**Published:** 2023-10-11

**Authors:** Jovanka Lukić, Gergő Gyalog, Zoltán Horváth, Anita Annamária Szűcs, Tijana Ristović, Amarela Terzić-Vidojević, Zsuzsanna J. Sándor, Uroš Ljubobratović

**Affiliations:** 1 Laboratory for Molecular Microbiology (LMM), Institute of Molecular Genetics and Genetic Engineering (IMGGE), University of Belgrade, Vojvode Stepe 444a, 11042 Belgrade, Serbia; lukicjovanka@imgge.bg.ac.rs (J.L.); amarela@imgge.bg.ac.rs (A.T.-V.); 2 Research Centre for Fisheries and Aquaculture, Institute of Aquaculture and Environmental Safety, Hungarian University of Agriculture and Life Sciences (MATE HAKI), Anna-Liget Str. 35, H-5540 Szarvas, Hungary; gyalog.gergo.sandor@uni-mate.hu (G.G.); szucs.anita.annamaria@uni-mate.hu (A.A.S.); ristovic.tijana@uni-mate.hu (T.R.); jakabne.sandor.zsuzsanna@uni-mate.hu (Z.J.S.); 3 H&H Carpió Halászati Kft., Kossuth u. 7, H-7814 Ocsard, Hungary

**Keywords:** largemouth bass, indoor, juveniles, nutrition, monounsaturated fatty acids (MUFA), ω3 polyunsaturated fatty acids (PUFA), probiotic, deformities, survival

## Abstract

**Simple Summary:**

This study aimed to evaluate the efficacy of complete commercially available feeds with varying nutritive profiles in the diet of juvenile largemouth bass (LMB) reared in a closed tank system. LMB is a lean fish rich in health-promoting ω3 fats. It is a promising new species for aquaculture worldwide, given its lack of intramuscular bones and delicious taste. Conditions for rearing LMB in tank systems as well as its nutritive demands from the early life stage have not yet been defined. The results of this study revealed monounsaturated (MUFA) and polyunsaturated (PUFA) ω3 fatty acids to be important nutrients for juvenile LMB, with MUFA possibly outbalancing the potential negative effects of oxidative stress caused by membrane-stabilizing ω3 fats. In addition, this study suggested an interesting feature of juvenile LMB: to sacrifice the development of some parts of the skeleton (possibly scales) for rapid growth. Among the tested feeds, one was selected as optimal for tank-reared juvenile LMB.

**Abstract:**

This study aimed to evaluate different commercial diets (Otohime C1, Aller Futura (AF), Biomar Inicio Plus (BIP)) and one experimental feed (EF) in terms of their effectiveness as post-larval diets for indoor weaned largemouth bass, LMB (*Micropterus salmoides*). Key variations in the content of nutritive values were monounsaturated fatty acid (MUFA) and highly unsaturated FA (HUFA) ω3. Fish were fed with one of four tested diets from the 33rd to the 40th day post-hatch (DPH). Biometric indices, digestive enzyme-specific activities, thyroid hormone status, and mRNA expression of genes coding for skeleton, neuron, and muscle growth were analyzed. The lowest skeletal deformity rate and highest survival among the treatments were seen in BIP-fed fish. Dietary lipids, with an appropriate balance between MUFA and polyunsaturated FA (PUFA), alongside amino acid balance, were shown to be the main contributors to the growth of the skeleton and/or fish survival. On the other hand, fish growth is correlated with fish digestive capacity and feed moisture percent rather than feed quality. Unexpectedly, BIP-fed fish were attributed with the lowest expression of skeleton differentiation markers, which may reflect the sacrifice of scale and/or cranium growth at the expense of somatic growth. This study highlights the role of non-marine ingredients in the nutrition of post-larval LMB.

## 1. Introduction

Largemouth bass, LMB (*Micropterus salmoides*), is a freshwater carnivorous fish species native to the American continent but introduced as a farmed species for human consumption on the Asian market at the end of the last century. LMB is an attractive new species for European aquaculture, given its delicious taste and the lack of intramuscular bones [1]. Furthermore, it contains high amounts of highly unsaturated fatty acids (HUFA), including docosahexaenoic acid (DHA) and eicosapentaenoic acid (EPA) [2].

A critical issue related to the cultivation of LMB is larviculture, which is commonly carried out in ponds until six to seven weeks after hatching, followed by feed training in the Recirculating Aquaculture System (RAS) for up to two weeks [3,4]. After feed training, fish are stocked back in the pond at the age of approximately two months for the grow-out [5]. This raises several issues in terms of economic viability: first, the vast space occupied, and second, the shortened grow-out and fattening phases. In addition, a recent study has shown that indoor larviculture of pike-perch may bring significant benefits for its growth in later life stages [6]. Thus, the establishment of indoor LMB larviculture can be deemed critical for the introduction of LMB farming in Europe.

After weaning indoors, the next phase is the on-grow phase in RAS to prepare the larvae for the grow-out diets in the pond. During traditional LMB culture, high-marine ingredient feed, Otohime C1, is commonly used for feed training after the pond-nursing phase [7]. Then, after stocking back to the pond for the grow-out, salmonid diets are used [3]. Nevertheless, careful evaluation of the nutritive demands of LMB during the on-growth phase may guide the selection of diets in later rearing stages (grow-out and further on), when higher feed quantities are used and sustainability issues are gaining importance. Related to this, vegetable monounsaturated FA (MUFA)-rich oils (e.g., canola oil) were shown to be a good alternative to fish oil. The beneficial effects of MUFA are largely attributed to its ability to be efficiently used as energy substrates and thereby spare HUFA [8,9]. In this line, this study aimed to evaluate commercially available on-grow diets with various inclusions of MUFA and polyunsaturated FA (PUFA) for LMB early juveniles nursed in RAS. An emphasis was placed on digestive capacity and the growth of skeletal, muscle, and neural tissue. Aside from MUFA and PUFA ω3 and ω6, the interplay between other nutritive elements was also evaluated to understand the nutritional needs of LMB, which has been insufficiently investigated, especially after larviculture in RAS. The knowledge obtained in this study could be used both by researchers and hatcheries, but it could also be applied to the development of custom-made diets for indoor-reared juvenile LMB.

## 2. Materials and Methods

### 2.1. Feeds Used in the Study

Three different commercial diets (Otohime C1, Aller Futura (AF), and Biomar Inicio Plus (BIP)) were selected from the market and tested in this trial (Table 1). Parallel with this, locally produced homemade food was prepared as an experimental diet. For this purpose, all the ingredients presented in Table 1 were homogenized with a 50-type mixer and extruded using a 0.5-mm-diameter die in an SGP-45 stainless steel extruder (Xingtai Tianruo Machinery Manufacturing Co. Ltd., Xingtai, China). After extrusion, the pellets were chopped up using the SSLG15-80 crumbler and kept at room temperature (RT) until use.

Proximate compositions of the diets were estimated by methods of the Hungarian Standard (dry matter MSZ ISO 6496:2001; crude protein MSZ EN ISO 5983-2:2005; crude fat 152/2009 EK III/H; crude fiber 152/2009 EK III/I; crude ash MSZ 5984:1992_withdrawn) [10,11,12,13,14]. The amino acid and fatty acid (FA) profiles were determined by using the Hungarian standards MSZ EN ISO 13903:2005 and MSZ EN ISO 12966-2, respectively [15,16].

### 2.2. Fish Origin

The animal research was approved by the Institutional Animal Welfare Committee of the Hungarian University of Agricultural and Life Sciences, Szent István Campus (MATE-SZIC/416-1/2022). Experiments were carried out in accordance with the “Animal research: reporting of in vivo experiments (ARRIVE)” guidelines and EU Directive 2010/63/EU. Juveniles for this study were obtained from a seasonal semi-artificial reproduction of largemouth bass. Namely, in the first week of May 2023, 30 pairs of breeders were selected from the broodstock rearing pond and treated with 50 μg·kg^−1^ salmon gonadotropin-releasing hormone analog (sGnRHa, [D-Arg6, Trp7, Leu8, Pro9-NEt]-GnRH, Bachem, Bubendorf, Switzerland). Later, fish were stocked in the spawning pond, which was equipped with 30 nests made out of artificial grass measuring 60 × 60 cm. Three days after hormonal treatments, 10 nests with fertilized eggs were removed from the spawning ponds, and eggs and newly hatched larvae were further incubated in the hatchery at a temperature of 23 ± 1 °C. Upon swim bladder inflation, 30,000 larvae were volumetrically counted and stocked in the cylindroconical larval rearing tank of water volume 1.4 m^3^ within the larval rearing RAS. The larval rearing system was composed of a total of 16 tanks, four of which were 1.4 m^3^ and 12 of which were 0.25 m^3^, all with the same design with a white conical bottom and black walls where water flow was maintained in an up-welling manner throughout the study. Upon stocking, larvae were fed for 10 days with enriched *Artemia* nauplii (SEP-ART GSL, INVE Technologies, Dendermonde, Belgium). Later, larvae were subjected to the weaning to dry diet of Otohime B2 feed (Marubeni Nisshin Feed Co., Ltd., Tokyo, Japan) for 14 days in total. Upon weaning, juveniles were harvested from the larger tanks, and 60 randomly chosen juveniles were stocked in each of the 12 smaller volume tanks.

### 2.3. Study Design

On the 33rd day post-hatch (DPH), weaned juveniles (average standard length 25.6 mm and average individual weight 195.2 mg) were harvested from the tank, and 60 randomly chosen juveniles were stocked in each of the 12 smaller volume tanks for an 8-day-long feeding trial. Feeding treatments were assigned randomly to each tank. Each tank received 6 g of feed per tank during the first two days (33, 34 DPH), then four days (35, 36, 37, and 38 DPH), 8 g per tank, and then, for the last two days (39 and 40 DPH), 9 g per tank. In the first three days, fish received a 50:50 mixture of the weaning diet, B2 and the respective treatment diet, while from the 4th day until the end of the trial, only the trial feed was supplied to the fish. Both larvae and juveniles were reared under the 16:8 LD photoperiod, and the feeding period matched the light period. Food was supplied every 5 min using the automatic belt feeder. Tanks were cleaned once a day, and mortalities were counted.

**Figure 1:**
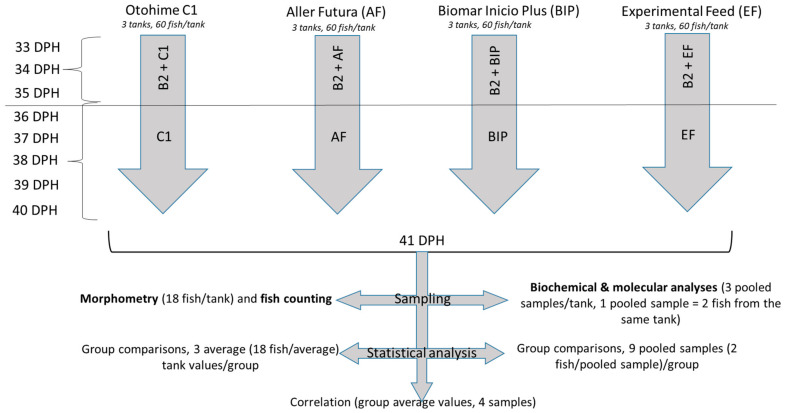
Chart showing the research workflow. B2 = Otohime B2, DPH = Day post-hatch.

The sampling of fish was carried out on the 41st DPH after 12 h of food restriction. Fish were anesthetized using phenoxyethanol (0.4 mL × L^−1^). Fish were dipped onto a paper towel to remove water drops, and morphometric measurements were performed on a representative sample of 18 fish per tank. For molecular and biochemical analyses, six fish per tank were sampled and stored at −80 °C until further use. The experimental workflow is presented in Figure 1.

### 2.4. Homogenization of Fish

For the isolation of RNA, whole fish (two fish per homogenization, totaling 700–800 mg) were homogenized in mortar using liquid nitrogen. After powdering the samples, denaturing buffer was added to the homogenate, and the homogenate was mixed with one volume of acid phenol, 1/10 volume of Na-acetate, pH 4, and 1/5 volume of chloroform [17]. After centrifugation (15,500× *g*, 15 min, 4 °C), the upper phase was moved to another tube, and the phenol extraction step was repeated. The extracted samples were precipitated using isopropanol and 70% ethanol, then dried at RT and suspended in distilled water. RNA concentration was measured using Nanodrop 2000 (Thermo Fischer Scientific, Waltham, MA, USA).

For biochemical analyses, samples (two fish per homogenization) were homogenized after the addition of 10 volumes of homogenization buffer using a dounce tissue grinder [17]. After 20–30 strokes with pestle A, homogenates were filtered using a Falcon^®^ 70 µm Cell Strainer (Corning Inc., Corning, NY, USA). Filtrate was additionally homogenized using Pestle B. Homogenates were freeze-thawed once to further lyze the cells and centrifuged at 4 °C. 15,500× *g*, for 15 min. The supernatants were transferred to another tube and stored at −20 °C until further use. Buffers used for homogenization were phosphate buffered saline (PBS) for ELISA and 50 mM Tris, pH 7, and 2 mM mannitol for digestive enzyme assays [17].

### 2.5. Quantitative PCR (qPCR)

Reverse transcription (RT) was performed using the Xpert cDNA Synthesis Kit (GRiSP Lda., Porto, Portugal), as suggested by the manufacturer, using 1 μg of RNA template. The reaction was performed in the SuperCycler Thermal Cycler SC3005 (Kyratec, Mansfield, Australia). Quantitative PCR (qPCR) was performed using Xpert Fast SYBR 2X Mastermix (GRiSP Lda., Porto, Portugal), using a five-fold dilution of complementary (c) DNA and the final 10 mM primer concentration in the mixture. Cycling conditions were:

1 × (95 °C, 2 min), 40 × (95 °C, 5 s, and 60 °C, 30 s)

Primer sequences (Table 2) were designed for the purpose of this study (SOX3). The design of the primers was performed using mRNA sequences available from the National Center for Biotechnology Information (NCBI) (https://www.ncbi.nlm.nih.gov (accessed on 28 August 2023)) and Primer-BLAST, NCBI. The GAPDH gene was an endogenous control. One sample from the C1 group was used as the reference for the Ct calculation. NCBI Gene Identification (ID), product length in base pairs (bp), and primer efficiency slopes (obtained using cDNA from this study as a template) are provided in Table 2.

### 2.6. Biochemical Analyses

Trypsin, chymotrypsin, lipase, amylase, and alkaline phosphatase (AP) activities were estimated as previously described [17], while the phospholipase A2 (PLA2) assay was performed using the Abcam PLA2 Assay Kit (Abcam, United Kingdom), following the manufacturer’s instructions [17]. For the trypsin, chymotrypsin, lipase, and AP assays, 10 μL of the sample was mixed with 190 μL of the reaction buffer containing adequate substrate: Nα-benzoyl-DL-arginine-ρ-nitroanilide hydrochloride (BAPNA) for the trypsin, succinyl-(ala)2-pro-phe-ρ-nitroanilide (SAPNA) for the chymotrypsin, p-nitrophenyl phosphate (p-NPP) for the AP, and ρ-nitrophenyl palmitate (ρ-NPP) for the lipase assay [17]. After mixing the samples with the substrate containing buffer, the absorbance measurement (407 nm for the AP assay and 410 nm for the trypsin, chymotrypsin, and lipase assays) started immediately and lasted 10 min at 37 °C, with measurements of the absorbance every minute. Enzyme-specific activities were calculated using the formula:

Enzyme-specific activity (mU × mg^−1^ of protein) = ((ΔAsample − ΔAblank)/time (min)) × 1000 × total volume of the reaction mixture (mL))/(ε × volume of the sample (mL) × prot conc (mg×mL^−1^))

ΔA: difference in the absorbance between the two time points

ε: the extinction coefficient of the product of the reaction (8800—trypsin and chymotrypsin assays, 18,300—AP assay, 15,000 M^−1^ ×cm^−1^—lipase assay)

For the amylase assay, 50 μL of the sample was mixed with 50 μL of 1% starch solution. After incubation for 15 min, RT, 50 μL of stop solution was added to stop the reaction, and the absorbance was measured at 540 nm [17].

Thyroxine (T4) and tri-iodothyronine (T3) concentrations were estimated using BioMatic (Canada) ELISA kits, according to the manufacturer’s instructions. The concentration of proteins in the homogenates was measured using the Analyticon Biotechnologies kit. Obtained results are expressed as μg per mg of protein for trypsin, chymotrypsin, lipase, and AP; nmol ×min^−1^ activity per mg of protein for PLA2; mmol of maltose standard released per min per mg of protein for amylase; and ng per mg of protein for T3 and T4 assays.

### 2.7. Economic Analysis

In order to compare the economic performance of different feeds used in this study, a cost calculation was carried out by simulating the nutrition cost and larval nursing infrastructure operation costs (representing labor, energy, and capital costs) spent on rearing 1 g of juveniles. It was hypothesized that reaching a mean weight of 1 g is the ultimate aim of the indoor training phase, and the output is released for subsequent stocking in outdoor ponds. For the calculation, the following equations were used:

Total costs (€ × juvenile^−1^) = nutrition cost (€ × juvenile^−1^) + infrastructure costs (€ × juvenile^−1^)

Nutrition cost = Feed conversion ratio (FCR) × unit cost of feed (€ × g^−1^) × (Wf−Wi)

Infrastructure cost = [rental fee (€ × day^−1^ × culture unit^−1^) × rearing time (day)]/production capacity (juvenile × culture unit^−1^)

The rearing time was calculated as follows, assuming an exponential growth function:

Rearing time = [Log(Wf) − Log(Wi)]/k

where the final weight of the rearing phase (Wf) is 1 g, the starting weight (Wi) is 0.2 g, and the k parameter is identical to specific growth rate (SGR) values calculated and presented in Section 3. Feed prices were sourced from commercial feed traders, while the daily rental fee table of the MATE HAKI larval rearing unit was used as a proxy for infrastructure operation costs. Starting in 2023, the rental rate was 21.3 € × day^−1^ for the system of 4 m^3^. Given the limited experience in LMB fry rearing, a rearing density of one juvenile per liter was allowed in the calculations. The cost of juveniles weighing 0.2 g, considered to be equal among treatment groups, was not accounted for in the analysis since the ultimate aim was to compare the economic performance between feeds.

### 2.8. Data Analysis

Biometric evaluation results are presented in tables as means ± standard deviations (S.D.) of the average values of three tanks (n = 3, one tank = the average value of the representative sample of 18 fish). Calculations of Specific Growth Rates (SGR), allometric coefficient (b), relative condition factor (Kn), and FCR were carried out as explained previously [6]. Survival and skeletal deformity rates were calculated as the percent of surviving and deformed fish out of the initial 60 fish per tank. The analysis was conducted using the Kruskal–Wallis (KW) test (https://www.statskingdom.com/kruskal-wallis-calculator.html (accessed on 28 August 2023)) (normality assumption and variance homogeneity could not be reliably evaluated with three samples per group, so ANOVA could not be applied).

The results of the biochemical and molecular analyses are provided as clustered bars showing means ± S.D. of pooled samples (two fish per one pooled sample, three pooled samples per tank, n = 9 pooled samples). Statistical analysis was performed using Analysis of Variance (ANOVA), with Tukey post-hoc if the distribution of data was normal and the variance homogenous, according to the Shapiro–Wilk and Levene’s test, after the removal of single outliers (if any) using Grubbs’ test (https://contchart.com/outliers.aspx (accessed on 28 August)). If normality assumption and variance homogeneity could not be achieved with non-transformed and log-transformed data, the analysis was performed using the KW test with non-processed data. Pearson correlation analysis was carried out between the group average values of all evaluated variables, including feed nutritive profile. Partial correlation analysis (Pearson test), controlled for MUFA level, was used to analyze the relationship with the PUFA ω3/ω6 ratio. The same was carried out for MUFA, controlled for PUFA ω3/ω6 ratio. The correlation with the presence or absence of Bactocell^®^ probiotic (dichotomous variable) was analyzed using the point-biserial correlation coefficient (http://www.vassarstats.net/pbcorr.html (accessed on 28 August 2023)). The results of the statistical analysis were considered significant if *p* < 0.05 and insignificant if a statistical trend (*p* < 0.1) was observed. Differences with *p* > 0.1 were not considered. Shapiro–Wilk and Levene’s test, Pearson correlation analysis, ANOVA, as well as table and graph drawing, were conducted using IBM SPSS Statistics 21 [18]. A visual representation of the correlation matrix was made using *corrplot* R package [19].

## 3. Results

### 3.1. Feed Composition

The proximate compositions of the feeds are presented in Table 3. The protein content of the feeds varied between 50.9 and 55.9%, with the lowest level in the EF diet. The crude fat content was altered within the treatments, and similarly, the lowest value was found in the EF diet. As a consequence of this, the amount of nitrogen-free extract was the highest in EF feed. The gross energy was in the range of 17.0–18.2 MJ × kg^−1^.

In terms of the FA composition (Table 4), as was expected based on the list of ingredients, the C1 diet presented the highest level of polyunsaturated FA (PUFA) ω3 (31.07%), while the EF diet contained the highest amount of PUFA ω6 (13.3%), due to the high level of linoleic acid (18:2 ω6). Regarding saturated FA (SFA), the C1 diet had the highest total level (36.11%), while the BIP diet had the lowest level (25.84%). Monounsaturated FA (MUFA) levels were the highest in the BIP diet (45.94%). Bivariate correlation analysis among total FA levels (PUFA ω3, PUFA ω6, MUFA, and SFA) revealed a negative correlation trend (*p* = 0.067) between total MUFA and SFA levels (correlation coefficient, r = −0.933). The percent of FA expressed per feed weight is provided in Supplementary File S1 (“Feed fatty acids per weight”).

Based on the amino acid measurements (Table 5), the sum of essential amino acids (EAA) varied between 22.3 and 24.5% without tryptophan, which was not quantified using this method. The levels of the main limiting essential amino acids, lysine and methionine, were adequate for LMB post-larvae, ranging from 3.9 to 4.3% and 1.51 to 1.59%, respectively [20,21]. The EF diet had higher lysine and lower arginine and valine content compared to C1.

### 3.2. Biometric Indices

Fish administered BIP presented a lower deformity (*p* = 0.03) and higher survival (*p* = 0.02) percent in comparison to the EF group (Table 6). No significant differences among the groups were observed for other parameters.

### 3.3. mRNA Expression

**Figure 2:**
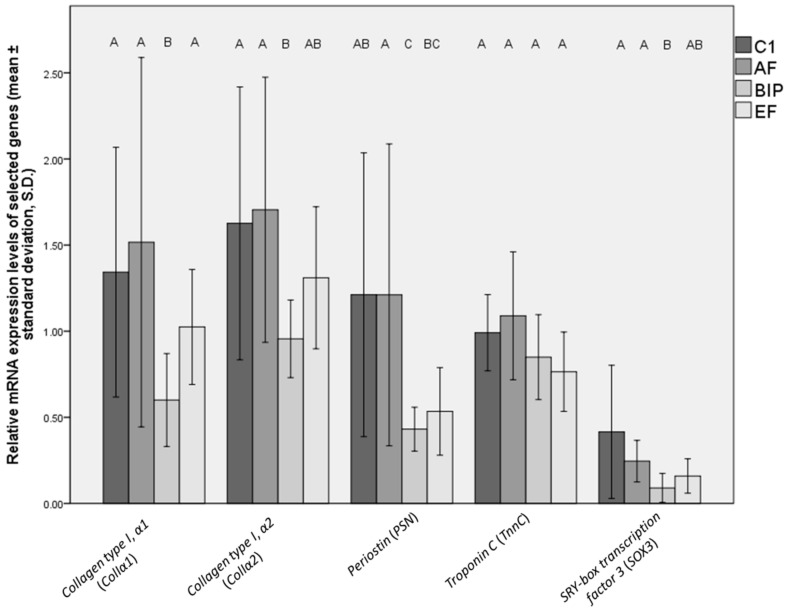
Relative gene (mRNA) expression levels in the whole bodies of C1, AF, BIP, and EF-fed fish, provided as mean values ± standard deviations (S.D.). Sample 1 from the C1 group was used as the calibrator; letters indicate statistically significant differences between the treatments (common letters indicate no significant difference). A comparison between the groups was carried out using the Kruskall–Wallis (KW) test. C1 = Otohime C1, AF = Aller Futura, BIP = Biomar Inicio Plus, EF = Experimental Feed.

BIP-administered fish showed lower mRNA expression levels of *ColIα1*, *ColIα2* and *PSN* in comparison to C1 (*p* = 0.009, 0.03, and 0.02, respectively) and AF (*p* = 0.008, 0.005, and 0.004), as well as the lower expression of *ColIα1* in comparison to EF (*p* = 0.04) (Figure 2). On the other side, the EF group was presented with significantly lower *PSN* mRNA expression in comparison to the AF group (*p* = 0.049). Similar to skeleton differentiation marker gene expression, the level of neurogenesis marker *SOX3* was lower in BIP-fed fish in comparison to both C1 (*p* = 0.02) and AF groups (*p* = 0.01).

### 3.4. Digestive Enzyme Activity

**Figure 3:**
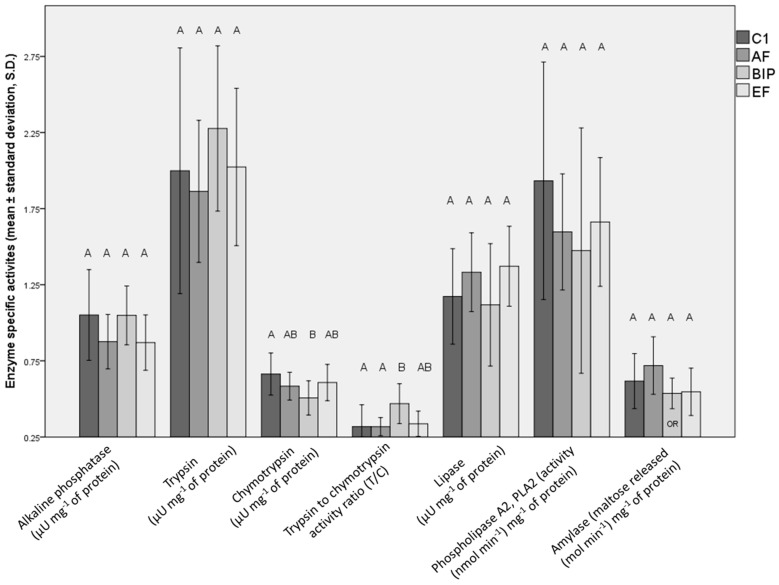
Digestive enzyme specific activities in the whole bodies of fish fed C1, AF, BIP, and EF, provided as mean values ± standard deviations (S.D.). Values of alkaline phosphatase activity and trypsin to chymotrypsin activity ratio (T/C) are presented as 10-fold lower values. Letters above the graphs indicate statistically significant differences between the treatments (common letters indicate no significant difference). Differences between the groups for alkaline phosphatase, trypsin, chymotrypsin, lipase and amylase specific activity were analyzed using the Kruskal–Wallis (KW) test, while differences between the groups for phospholipase (PLA2) activity and T/C were estimated using ANOVA. OR = outlier removed, C1 = Otohime C1, AF = Aller Futura, BIP = Biomar Inicio Plus, EF = Experimental Feed.

At the level of digestive enzyme-specific activities (Figure 3), differences were seen only in chymotrypsin-specific activity, with C1-fed fish having higher activity in comparison to the BIP group (*p* = 0.036). The trypsin-to-chymotrypsin (T/C) activity ratio was significantly higher in the BIP group in comparison to C1 (*p* = 0.031) and the AF group (*p* = 0.031).

### 3.5. Hormonal Status

**Figure 4:**
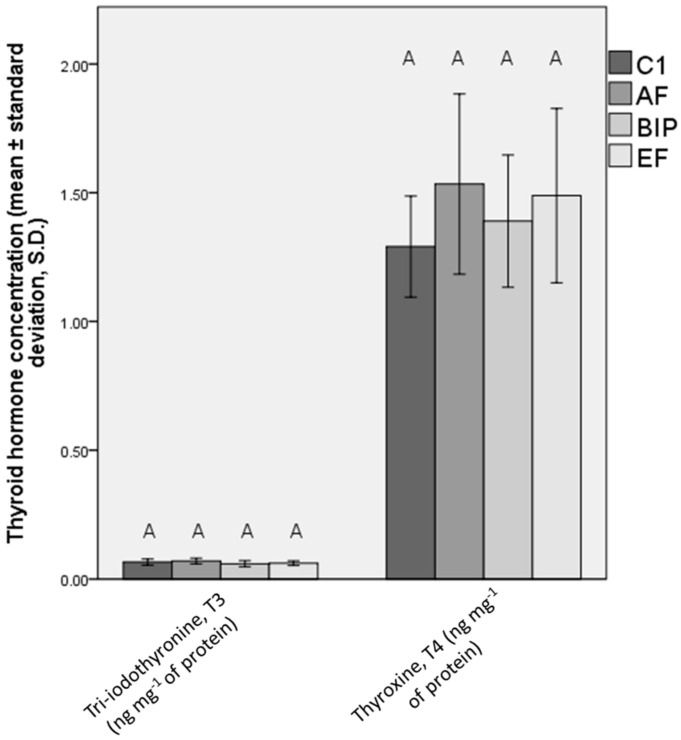
Concentrations of T3 and T4 in the whole bodies of fish fed C1, AF, BIP, and EF, provided as mean values ± standard deviations (S.D.). Letters indicate statistically significant differences between the treatments (common letters indicate no significant difference). Differences between the treatments were analyzed using the Kruskall–Wallis (KW) test. C1 = Otohime C1, AF = Aller Futura, BIP = Biomar Inicio Plus, EF = Experimental Feed.

Thyroid hormone levels are presented in Figure 4. No significant differences in the levels of thyroid hormone levels were seen between the treatments.

### 3.6. Correlation Analysis

**Figure 5:**
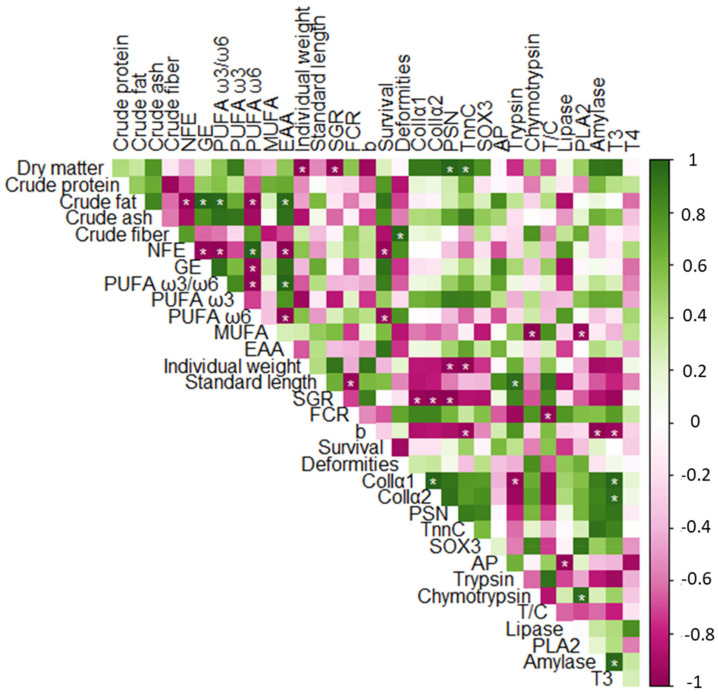
Visual representation of bivariate correlation matrix showing the association between dietary nutritive value, including dry matter percent (%), crude protein %, crude fat %, crude ash %, crude fiber %, nitrogen free extract (NFE) %, gross energy (GE, MJ × kg^−1^), polyunsaturated fatty acid (PUFA) ω3/ω6 ratio, PUFA ω3 %, PUFA ω6 %, monounsaturated fatty acid (MUFA) % and essential amino acid (EAA) %, and fish performance indicators, including individual weight (mg), standard length (mm), specific growth rate (SGR, % × day^−1^), feed conversion ratio (FCR), allometric coefficient (b), survival %, deformity %, mRNA expression level of *collagen type I*, *α1* (*ColIα1*), *ColIα2*, *periostin* (*PSN*), *troponin C* (*TnnC*), *SRY-box transcription factor 3* (*SOX3*), specific activities of alkaline phosphatase (AP, μU ×mg^−1^ of protein), trypsin (μU × mg^−1^ of protein), chymotrypsin (μU × mg^−1^ of protein), trypsin to chymotrypsin activity ratio (T/C), lipase (μU × mg^−1^ of protein), phospholipase (PLA2, activity (nmol ×min^−1^) ×mg^−1^ of protein) and amylase (maltose released (mmol × min^−1^) × mg^−1^ of protein), as well as the level of thyroid hormones tri-iodothyronine (T3, ng × mg^−1^ of protein) and thyroxine (T4, ng × mg^−1^ of protein). * indicates significant correlations (*p* < 0.05).

Bivariate correlation analysis (Figure 5) revealed a positive association of fish weight with feed dry matter percent and a negative correlation with *PSN* and *TnnC* mRNA expression. Standard length correlated positively with trypsin-specific activity and negatively with FCR. SGR showed a negative correlation with dry matter percent in the diet, as well as *ColIα1*, *ColIα2* and *PSN* mRNA expression levels. FCR showed a negative correlation with standard length and the trypsin-to-chymotrypsin (T/C) activity ratio, while the allometric coefficient correlated negatively with *TnnC* mRNA expression, amylase specific activity, and T3 level. Fish survival correlated negatively with NFE and PUFA ω6 in the diet, while fish deformity rate showed a positive correlation with crude fiber percent in the diet. *ColIα1/ColIα2* mRNA expression levels correlated positively with *ColIα2/ColIα1* mRNA and T3 level and negatively (*ColIα1*) with trypsin specific activity. *ColIα1*, *ColIα2,* and *PSN* mRNA expression levels were negatively correlated with SGR. Both *PSN* and *TnnC* mRNA expression showed a positive correlation with feed dry matter percent. Alkaline phosphatase specific activity correlated negatively with lipase specific activity. Chymotrypsin specific activity showed a negative correlation with MUFA percent in the diet and a positive correlation with PLA2 specific activity. Similarly, PLA2 specific activity showed a negative correlation with MUFA percent in the diet (with or without correction for the PUFA ω3/ω6 ratio). Amylase specific activity was positively related to T3 levels. The condition factor, Kn, was not included in the correlation analysis, given the low differences in Kn values between the groups (second decimal).

Partial correlation analysis, controlling for the PUFA ω3/ω6 ratio, revealed a significant correlation of MUFA percent with fish survival. Likewise, partial correlation analysis, controlling for MUFA percent, demonstrated a significant correlation of the PUFA ω3/ω6 ratio with survival. Point biserial correlation analysis revealed a negative correlation between probiotic presence in the diet and FCR (r_pb_ = −0.99, *p* = 0.006). In addition, probiotic presence correlated positively with the T/C activity ratio (r_pb_ = 0.99, *p* = 0.01).

The values of correlation coefficients and *p* values for the above-detailed bivariate and partial correlations are provided in Supplementary File S2 (“Correlation coefficients”).

### 3.7. Economic Calculations

Costs related to juvenile LMB production using the feeds tested in this research are shown in Table 7. The economic analysis showed that fish fed commercially available feed manufactured in Europe (AF and BIP) had a notable cost advantage over fish fed with imported feed (C1) and experimentally produced feed (EF).

## 4. Discussion

This research evaluated the performance of four complete diets as on-grow feeds for indoor nursed largemouth bass (LMB), previously weaned using Otohime B2. Tested feeds included three commercial diets: Otohime C1 (C1), Aller Futura (AF), Biomar Inicio Plus (BIP), and one homemade experimental feed (EF). The selection of the diets was based on two critical parameters: highly polyunsaturated fatty acid (PUFA) ω3 (high in C1, low in EF), and monounsaturated fatty acid (MUFA) level (high in BIP and low in C1). Namely, the high PUFA ω3 content in C1 feed most probably reflects a high marine ingredient percent in the diet [22]. On the other hand, MUFA, along with the MUFA/saturated FA (SFA) ratio and linoleic acid (LA) content (high and low in BIP, respectively), are considered to be indicators of the presence of non-ω6 vegetable fats [23,24]. The above two parameters are critical when evaluating both the sustainability and prospective efficacy of fish feeds.

BIP-fed fish showed the lowest skeletal deformity rate. One of the key nutritive factors affecting skeletal development in fish is highly unsaturated FA (HUFA) level, precisely the specific balance between ω3 and ω6 HUFA in tissue, namely (the docosahexaenoic acid (DHA) + eicosapentaenoic acid (EPA))/arachidonic acid (ARA) ratio [25]. HUFA is believed to affect gene expression through interaction with the Peroxisome Proliferator Activated Receptor (PPAR) [26]. Expression of genes involved in the formation of the bone matrix is the prerequisite for mineralization, which provides firmness to the skeleton, but may be incomplete in early fish juveniles [27]. HUFA ω3/ω6 balance is affected by the total amount of ω3 and ω6 PUFA in the feed, so, aside from DHA, EPA, and ARA levels in the diet, levels of LA and alpha-linolenic acid (ALA) matter as well, since LA and ALA are involved in the synthesis of ω6 and ω3 HUFA, respectively. Nevertheless, excess dietary levels of HUFA have been paradoxically linked with skeletal malformations and muscle damage. This can be attributed to an increase in oxidative stress, since the addition of α-tocopherol (vitamin E) is able to reverse this trend [28,29]. Vitamin E is commonly found in MUFA-rich fats, mostly vegetable oils [30,31]. In addition, independently of the vitamin E, MUFA were shown to reduce cellular oxidative stress by reducing membrane lipid desaturation while still maintaining membrane fluidity [32]. Due to the common mechanism of action of MUFA and HUFA ω3/ω6 (integration into cellular membranes) inside the living system, a partial correlation analysis of MUFA and PUFA ω3/ω6 with analyzed physiological parameters was performed, in addition to the standard bivariate correlation analysis. MUFA levels in the diets showed a negative correlation trend with skeletal deformity rate (*p* = 0.07) when corrected for the PUFA ω3/ω6 ratio. A similar negative correlation trend was noticed between the PUFA ω3/ω6 ratio and the skeletal deformity rate (*p* = 0.09) when adjusted for the MUFA level. In line with this, the highest skeletal deformation rate was seen in fish fed EF, which showed the lowest MUFA and PUFA ω3/ω6 ratio. It appears that both factors contribute to skeletal development and that an appropriate balance between tissue MUFA and HUFA ω3 and ω6 may be critical for the adequate formation of skeletal elements. Correlation analysis additionally revealed the negative role of dietary fibers in skeleton development. Indigestible dietary fibers were shown to retard the absorption of nutrients by reducing the transit time of digestion [33]. This might have caused the deficiency of essential nutrients needed for skeleton development in fish fed high-fiber diets.

Although the PUFA ω3/ω6 ratio in this research has shown a negative correlation with the skeletal deformity rate, it is applicable only in the range of PUFA present in the diets in this research. This is because the results on the ARA role in skeleton growth are somewhat contradictory, given the important role of the ARA metabolite, prostaglandin E2 (PGE2), in skeleton remodeling [34,35]. Furthermore, LA, along with oleic acid (OA), which was similarly increased in EF, can inhibit the synthesis of ARA when in excess [36]. Additional research in this direction would be needed to delineate the exact amounts of LA, OA, ALA, and HUFA in the diet to support the development of the skeleton of post-larval LMB.

Skeletal deformities are usually accompanied by high mortality, as also revealed in the present study [26]. Similar to skeleton quality, survival was positively related to MUFA (PUFA ω3/ω6 controlled) and the PUFA ω3/ω6 ratio (MUFA controlled). In addition, positive correlation trends between fish survival, on one side, and crude fat percent (*p* = 0.08) and essential amino acid level (*p* = 0.072) (low in EF) in the diet, on the other side, were observed. This was accompanied by a negative correlation with soluble carbohydrate levels in the diet (high in EF). The above results suggest that feed quality is the main determinant of fish survival. These results, furthermore, indicate carnivorous feeding habits of post-larval LMB, supporting previous studies on the negative influence of dietary carbohydrates on the performance of LMB [37].

Specific growth rate (SGR) and individual fish weight appeared to be positively related to the moisture percent of the diets (low in C1 and AF). High dry matter percent, although beneficial in terms of feed storage stability, may potentially increase the energy needed for food disintegration, decreasing the overall energetic value of the feed. The negative correlation trend (*p* = 0.064) between trypsin-specific activity and feed conversion ratio (FCR) confirmed the well-known role of trypsin as an indicator of fish digestive capacity [38]. Furthermore, the presence of Bactocell^®^ in the BIP diet was negatively related to FCR and showed a positive correlation trend (*p* = 0.09) with trypsin-specific activity, indicating its role in improving fish digestive capacity. Bactocell^®^ is the trade name for *Pediococcus acidilactici* CNCM I-4622, which was shown to improve fish skeletal development and growth rate in salmonids [39]. In general, Lactic Acid Bacteria (LAB) have been associated with the stimulation of the growth and skeletal differentiation of fish larvae in numerous species [40,41]. Potential mechanisms may include the improvement of nutrient availability in the case of growth and the reduction of inflammation and/or oxidative stress in the case of the latter [42,43]. Although no correlation between FCR and SGR was observed, FCR correlated inversely with fish length, which, on the other side, correlated positively with trypsin-specific activity. Length gain is an indicator of fish growth, presumably elongation of the axial skeleton, while weight gain reflects fat deposition as well [44]. Therefore, balancing feed moisture percent with the addition of probiotics may support optimal fish growth, including both skeleton growth and lipid deposition.

Although the BIP group showed the lowest skeletal deformity rate, the mRNA expression of ossification markers, *ColIα1* and *ColIα2*, was the lowest among the treatments. This has seemed contradictory since minerals, which give strength to the bones and prevent deformities, are deposited into the matrix made by collagen type I [45,46]. Hence, it would be very bold to assume that the development of the axial skeleton was slower in the BIP group. However, metamorphosis in teleost fish is marked by the appearance of scales, among other developmental events [47]. Scales are composed of collagen type I and minerals, so the expression of *ColIα1* and *2* mRNA in post-larval fish may come from both bones and scales, but from the skin as well [48,49]. Since a negative correlation was observed between *PSN*, *ColIα1* and *2* mRNA expression, on one side, and SGR, on the other side, it is possible that, in the face of a high growth rate, fish trade off the somatic growth with the differentiation of some tissues that may not be critical for survival in a specific environment. In support of our observation, lower scale strength in fast-growing individuals of pumpkinseed sunfish, *Lepomis gibbosus* (belonging to the same family as LMB, *Centrarchidae*), was observed in previous research [50]. Furthermore, the sacrifice of cranial skeleton ossification for fast growth was demonstrated in the same fish species [51]. It is thus possible that the lower expression of *ColIa1* and *ColIa2* mRNA seen in this research reflected both lower scale and cranial skeleton differentiation.

Expression of *SOX3* mRNA, the marker of neural tissue growth, was also low in BIP-administered fish [52]. A positive correlation trend (*p* = 0.074) between *SOX3* mRNA and PLA2 specific activity was observed, while overall MUFA percent in the diet appeared to suppress PLA2 activity. Although the polar and neutral lipid profiles of the diets were not investigated in this study, data from the literature suggest that neutral lipids are the preferred forms for MUFA deposition [53,54]. Therefore, the inverse association of MUFA with PLA2-specific activity may reflect the low phospholipid content of MUFA-rich feeds. Neural tissue growth is highly dependent on the availability of phospholipids in the diet, since phosphatidylcholine-bound FA are the preferred FA source for the brain, in comparison to free FA obtained after neutral lipid digestion [55]. Therefore, the BIP diet may be inferior in terms of fish brain development, particularly in comparison to the C1 feed.

Interestingly, C1-fed fish showed a higher chymotrypsin-specific activity in comparison to the BIP group. Chymotrypsin activity in fish was reported to suppress fish growth when growth conditions and/or food availability are suboptimal [56,57], which was probably the case in LMB post-larvae, given the low digestive capacity of fish in the early life stages [58]. Presumably, when feed quality is suboptimal, chymotrypsin may act as a regulator to reduce the flow of energy towards growth and direct it towards basic metabolic needs. In the present research, chymotrypsin specific activity is inversely correlated with MUFA percent in the diet. Given the positive association between PLA2 and chymotrypsin specific activity, it is possible that the activity of chymotrypsin is determined by the balance between polar and neutral lipids. Whatever the reason for decreased chymotrypsin specific activity in the BIP-fed group, the T/C activity ratio, which was increased in this group, showing a positive correlation trend (*p* = 0.09) with fish length, is deemed a reliable indicator of future (one–two month) fish growth [57]. Given the high SGR of BIP-fed fish during the experiment, the high T/C ratio probably does not reflect future compensatory growth but rather the higher growth potential of BIP-fed fish in comparison to other groups. Interestingly, this implies that FCR, which directly affects fish length, is a good indicator of future fish growth.

Expression of muscle growth marker mRNA, *TnnC*, was directly related to dry matter percent in the diet. In this line, *TnnC* mRNA showed a trend towards lower levels in the EF group in comparison to both C1 (*p* = 0.06) and AF (*p* = 0.05)-fed fish. It is possible that the low moisture percent in C1 and AF reduced the availability of the feed (through, e.g., longer floating time), imposing the need for intense swimming activity in order to reach the feed granules. This might have affected the muscle buildup, but it could also be the reason for the positive correlation trend between amylase-specific activity and the above two factors (*TnnC* mRNA expression (*p* = 0.069) and dietary dry matter percent (*p* = 0.078)), since carbohydrates are the most accessible substrates for rapid energy acquisition. In this line, high dry matter percent in the diet seemed to boost the overall metabolic rate, independent of *TnnC* expression, as evident from the positive correlation trend with T3 concentration (*p* = 0.061) [59].

**Figure 6:**
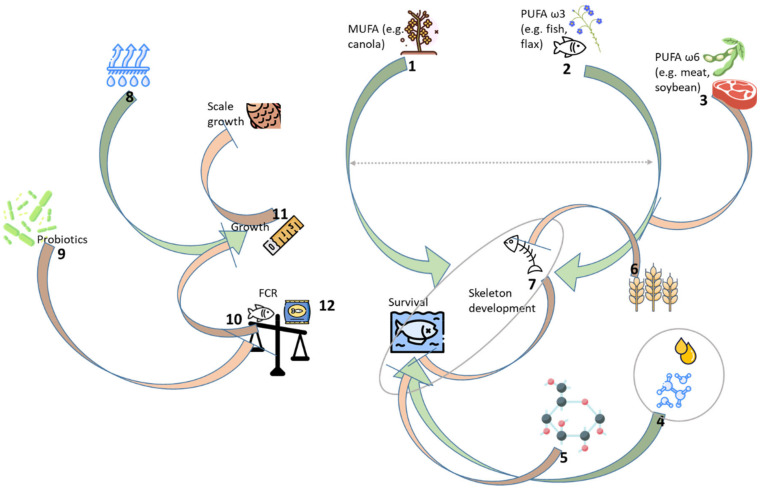
Assumed interactions among nutritive factors in the growth of post-larval largemouth bass (LMB) in the Recirculating Aquaculture System (RAS). Optimal balance between MUFA (1) and PUFA ω3 and ω6 (2, 3) in diets leads to improved skeleton growth. This MUFA/PUFA balance additionally affects fish survival, which is also influenced by feed fat content and protein quality (4), as well as the carbohydrate content of the diet (5). Skeletal development is negatively affected by the amount of dietary fiber (6), and it positively affects fish survival (7). On the other side, feed moisture percent improves fish growth rate (8), while the addition of probiotics to the diet improves FCR (9). FCR additionally affects fish growth (10). High growth rates can suppress the growth of body parts not highly relevant for survival in RAS (e.g., scales) (11); MUFA = monounsaturated fatty acids; PUFA = polyunsaturated fatty acids; and FCR = Feed Conversion Ratio. Icons used in the illustration have been downloaded from www.flaticon.com (accessed on 28 August 2023) (free license) and are used according to the Terms of Use provided at https://www.freepikcompany.com/legal#nav-flaticon (accessed on 28 August 2023). Icons were created by: *Freepik*, *Those Icons*, *Darius Dan*, *Nortch*, *max.icons*, *DinosoftLabs*, *surang*, rang, *Nendra Wahyu* and *Smashicons*.

From a biological standpoint, this research unequivocally demonstrated the superior performance of MUFA-rich BIP feed as the first juvenile feed for indoor-reared LMB, both in terms of survival and future growth potential. Although FCR in this study could not be precisely calculated, since fish were small and a common practice is to administer a large excess of feed to such small fish, feeds associated with larger SGR (BIP and EF) contributed to clearly lower per-unit rental fees owing to the shorter rearing period necessary to reach the target weight. An illustration of the key results of this study discussed above is provided in Figure 6.

## 5. Conclusions

This study underlines the role of vegetable MUFA and adequate MUFA-to-PUFA balance in the prevention of skeletal deformities in post-larval LMB. It also indicates the non-overlapping roles of factors influencing fish growth (energy balance of the diet and fish digestive capacity) and factors influencing fish skeletal development and survival (feed quality and total nutrient availability). Furthermore, this study showed the ability of LMB post-larvae to trade off the differentiation and/or growth of supporting tissues for rapid somatic growth. The consequences of this phenomenon, both beneficial in terms of improving fish growth in the long term and deleterious in terms of the adaptability of fast-growing individuals to grow-out pond environments, should be evaluated in further research. The knowledge presented here can support the selection of effective and sustainable diets for the on-growth of LMB, but it can also set the direction for the design of improved LMB diets aligned with the nutritional needs of LMB.

## Figures and Tables

**Table 1:** Raw materials in Otohime C1 (C1), Aller Futura Ex Gr (AF), Biomar Inicio Plus (BIP) and Experimental Feed (EF). The lists of ingredients for C1, AF and BIP have been provided by the manufacturers.

C1, 0.58–0.84 mm https://www.mn-feed.com (accessed on 28 August 2023)	Fish-meal, krill-meal, squid-meal, wheat flour, potato starch, fish oil, brewer’s yeast, vitamin and mineral premix, inorganic calcium phosphate, soy lecithin, guar gum, betaine, Paracoccus bacterial cell (astaxanthin) powder, calcium carbonate
AF, 0.5–1 mm https://www.aller-aqua.com (accessed on 28 August 2023)	Fish-meal, fish oil, grain products, krill-meal, single-cell proteins, vegetable proteins, vitamins and minerals, undisclosed functional ingredients
BIP, 0.8 mm www.aqua-garant.com (accessed on 28 August 2023)	Fish-meal, wheat, fish oil, krill-meal, hydrolyzed fish proteins, soya concentrate, monocalcium phosphate, yeast extract, minerals, astaxanthin, Pediococcus acidilactici CNCM I-4622 (Bactocell^®^)
EF, 0.5–1 mm This study	Fish-meal, pre-digested fish-meal, krill-meal, squid-meal, egg powder, chicken protein concentrate, brewer’s yeast, wheat flour, egg protein, liver-meal

**Table 2:** Primers used in the study.

*TnnC*	*Troponin C*, skeletal muscle mRNA	GAACCTTCCCTGATCGCCTT	fw
	Gene ID: 119906174, Product length: 99 bp, Slope: −3.0	GAGGAGGTGGGGCTTAAGTG	rev
*Col1α2*	*Collagen, type I, α 2* mRNA	TCTGAGAGGACTGAACGGACT	fw
	Gene ID: 119906985, Product length: 88 bp, Slope: −2.8	AACAAGGTGTTTTCCCGCGT	rev
*ColIα1*	*Collagen, type I, α 1a* mRNA	GCGGTGAGAGGAATGAAAGGA	fw
	Gene ID: 119882269, Product length: 98 bp, Slope: −3.16	TGGCTGTCAGTTTCACCGTT	rev
*PSN*	*Periostin*, osteoblast specific factor b mRNA	ACCAAACCCAGCCGTTGTAA	fw
	Gene ID: 119882985, Product length: 95 bp, Slope: −4.3	TTTGTCAGTTATACCTATTGCAGGA	rev
*SOX3*	*SRY-box transcription factor 3* mRNA	GAGAGGCTGGTGTGTTTCTGA	fw
	Gene ID: 119888700, Product length: 97 bp, Slope: −2.9	TTTGGACACAGTCGAGACAACT	rev
*GAPDH*	*Glyceraldehyde-3-phosphate dehydrogenase* mRNA	GCACTGTCACATCACCCACAT	fw
	Gene ID: 119897446, Product length: 86 bp, Slope: −3.7	TTCCTTCAGGCATCTAACAGGG	rev

**Table 3:** Proximate composition of C1, AF, BIP and EF.

	C1	AF	BIP	EF
Dry Matter (%)	93.2	93.5	91.6	91.6
Crude Protein (%)	52.0	55.9	54.3	50.9
Crude Fat (%)	17.6	14.9	18.4	8.3
Crude Ash (%)	12.8	12.9	11.8	9.0
Crude Fiber (%)	1.8	0.4	0.3	2.2
* Nitrogen Free Extract (%)	9.0	9.4	6.8	21.2
** Gross energy (MJ × kg^−1^)	18.0	17.7	18.2	17.0

C1 = Otohime C1, AF = Aller Futura, BIP = Biomar Inicio Plus, EF = Experimental Feed; * diets’ nitrogen free extracts (NFE) and gross energy (GE) values were calculated as NFE = 100 − (crude protein + crude fat + crude fiber + ash + moisture); ** GE (MJ × kg^−1^) = NFE × 23.6 + Protein × 17.2 + Lipids × 39.5, respectively.

**Table 4:** Fatty acid (FA) composition of C1, AF, BIP, and EF, provided as percent (%) of total FA in the respective feeds.

	C1	AF	BIP	EF
C12:0	0.09	0.04	0.05	0.20
C14:0	6.77	4.13	5.54	5.65
C14:l	0.09	0.05	0.07	0.08
Cl5:0	0.34	0.36	0.36	0.29
C16:0	23.36	18.01	17.13	22.57
Cl6:1	5.56	5.05	7.07	5.31
C17:0	0.16	0.24	0.20	0.19
C18:0	4.93	3.00	2.13	4.32
Cl8:1 ω9, OA	16.73	19.32	17.61	22.77
C18:2 ω6, LA	4.73	4.85	3.72	12.06
C18:3 ω6	0.16	0.12	0.14	0.15
C18:3 ω3, ALA	2.27	1.51	1.23	1.47
C20:0	0.15	0.23	0.22	0.26
C20:1	2.71	10.49	17.06	3.36
C20:2	0.18	0.39	0.29	0.26
C20:3 ω6	0.09	0.09	0.09	0.12
C20:4 ω6, ARA	0.57	0.71	0.49	0.71
C20:3 ω3	0.23	0.18	0.09	0.14
C20:5 ω3, EPA	14.74	10.79	9.53	9.03
C22:0	0.18	0.20	0.14	0.23
C22:1	1.11	1.49	2.15	0.80
C22:5 ω3	1.52	0.82	0.89	0.76
C24:0	0.13	0.1	0.09	0.17
C22:6 ω3, DHA	12.31	15.42	11.73	8.31
C24:1	0.88	2.42	1.98	0.80
SFA	36.11	26.31	25.86	33.88
MUFA	27.08	38.82	45.94	33.12
MUFA/SFA	0.75	1.48	1.78	0.98
PUFA ω3	31.07	28.72	23.47	19.71
PUFA ω6	5.73	6.16	4.73	13.30
PUFA ω3/ω6	5.42	4.66	4.96	1.48
(DHA + EPA)/ARA	47.5	36.9	43.4	24.4

C1 = Otohime C1, AF = Aller Futura, BIP = Biomar Inicio Plus, EF = Experimental Feed; OA = oleic acid, LA = linoleic acid, ALA = α-linolenic acid, ARA = arachidonic acid, EPA = eicosapentaenoic acid, DHA = docosahexaenoic acid, SFA = saturated FA, MUFA = mono-unsaturated FA, PUFA = polyunsaturated FA.

**Table 5:** Amino acid (AA) profiles of C1, AF, BIP, and EF diets, provided as the percent (%) per feed weight.

	C1	AF	BIP	EF
Arginine	3.31	3.22	3.31	2.82
Histidine	1.42	1.38	1.57	1.20
Isoleucine	2.41	2.77	2.32	2.38
Leucine	4.11	4.08	4.15	3.47
Lysine	4.22	3.93	4.21	4.31
Methionine	1.59	1.54	1.57	1.51
Phenylalanine	2.37	2.38	2.24	2.24
Threonine	2.25	2.34	2.28	2.04
Valine	2.78	2.73	2.85	2.33
ƩEAA	24.46	24.37	24.5	22.3
Alanine	3.49	3.20	3.41	2.95
Aspartic acid	5.32	5.31	5.26	4.52
Cysteine	0.49	0.41	0.45	0.44
Glutamic acid	9.86	8.09	9.01	8.91
Glycine	3.63	2.93	3.36	4.46
Proline	2.48	2.10	2.30	2.07
Serine	2.45	2.31	2.41	2.08
Tyrosine	1.78	1.91	1.79	1.56
ƩNEAA	29.50	26.26	27.99	26.99

C1 = Otohime C1, AF = Aller Futura, BIP = Biomar Inicio Plus, EF = Experimental Feed; ƩEAA = sum of essential amino acids, ƩNEAA = sum of non-essential amino acids.

**Table 6:** Morphometric indices of fish fed C1, AF, BIP, and EF provided as mean values ± S.D.; superscripts indicate statistically significant differences between the treatments (common letters indicate no significant difference); KW—Kruskal–Wallis.

	C1	AF	BIP	EF
Individual weight (mg) ^KW^	380 ± 25 ^A^	373 ± 42 ^A^	412 ± 19 ^A^	421 ± 63 ^A^
Standard length (mm) ^KW^	33.09 ± 1.53 ^A^	32.65 ± 1.32 ^A^	33.72 ± 0.22 ^A^	32.83 ± 1.47 ^A^
Specific growth rate (weight), SGR (% × day^−1^) ^KW^	6.96 ± 0.57 ^A^	6.94 ± 1.41 ^A^	8.31 ± 0.23 ^A^	7.95 ± 1.79 ^A^
Relative condition factor, Kn ^KW^	1.02 ± 0.03 ^A^	1.03 ± 0.04 ^A^	1.01 ± 0.01 ^A^	1.01 ± 0.00 ^A^
Feed conversion ratio, FCR ^KW^	6.30 ± 0.57 ^A^	6.50 ± 1.19 ^A^	4.90 ± 0.37 ^A^	6.45 ± 3.12 ^A^
Allometric coefficient (b) ^KW^	2.62 ± 0.76 ^A^	2.14 ± 1.38 ^A^	3.04 ± 0.29 ^A^	3.05 ± 0.07 ^A^
Survival (%) ^KW^	92.22 ± 2.55 ^AB^	93.33 ± 3.33 ^AB^	95.56 ± 0.96 ^B^	87.22 ± 5.85 ^AC^
Deformities (%) ^KW^	61.11 ± 3.47 ^AB^	57.22 ± 5.36 ^AB^	55.00 ± 3.33 ^B^	63.33 ± 1.67 ^AC^

C1 = Otohime C1, AF = Aller Futura, BIP = Biomar Inicio Plus, EF = Experimental Feed.

**Table 7:** Calculated per-unit production costs of 1 g juveniles * Rental fees stand for capital, labor, electricity, and water costs.

	C1	AF	BIP	EF
Nutrition costs (€ × juvenile^−1^)	0.0889	0.0151	0.0144	0.0929
Infrastructure rental fee * (€ × juvenile^−1^)	0.1229	0.1233	0.1029	0.1076
Total costs (€ × juvenile^−1^)	0.2118	0.1383	0.1173	0.2005

C1 = Otohime C1, AF = Aller Futura, BIP = Biomar Inicio Plus, EF = Experimental Feed.

## Data Availability

Raw data from this study are available at ResearchGate next to the Abstract of this article.

## Supplementary Materials

The following supporting information can be downloaded at: https://www.mdpi.com/article/10.3390/ani13203179/s1, Supplementary File S1: Feed fatty acids per weight, and Supplementary File S2: Correlation coefficients.
